# Reoccurring discogenic low back pain (LBP) after discoblock treated by oblique lumbar interbody fusion (OLIF)

**DOI:** 10.1186/s13018-020-1554-6

**Published:** 2020-01-20

**Authors:** Junhui Liu, Yongqing He, Bao Huang, Xuyang Zhang, Zhi Shan, Jian Chen, Shunwu Fan, Fengdong Zhao

**Affiliations:** 1 Department of Orthopaedics, Sir Run Run Shaw Hospital, School of Medicine, Zhejiang University, Key Laboratory of Musculoskeletal System Degeneration and Regeneration Translational Research of Zhejiang Province, No. 3, Qingchun Road East, Hangzhou, 310016 People’s Republic of China; 2 Department of Orthopaedics, Haining County People’s Hospital, Haining, People’s Republic of China

**Keywords:** Discogenic LBP, Discoblock, OLIF

## Abstract

**Objective:**

To determine the efficacy of OLIF in the treatment of reoccurring discogenic low back pain (LBP) after discoblock

**Methods:**

We included 108 patients with LBP that was suspected to be discogenic (such as high intensity zone, Schmorl’s nodes, Modic changes Type I, etc.), from August 2015 to August 2017. All patients underwent discography, and patients whose LBP was confirmed to be discogenic received discoblock. Patients who had reoccurring pain after discoblock underwent OLIF. Perioperative parameters and complications were recorded. The VAS and Oswestry Disability Index (ODI) were assessed at preoperation, and 1 week and 1, 3, 6, and 12 months after the surgery. The fusion rate was evaluated.

**Results:**

Of 108 patients, 89 were confirmed to have discogenic LBP, and 32/89 patients with reoccurring LBP pain after discoblock underwent OLIF. Twenty-eight patients were followed up for ≥ 1 year. The OLIF operation lasted for 92 ± 34 min. Blood loss during the operation was 48 ± 15 ml. The mean incision length was 3.0 ± 0.6 cm. The average length of stay was 4.8 ± 1.9 days. The VAS and ODI scores decreased from 8.1 ± 1.7 preoperatively to 0.9 ± 0.4, and from 71.2 ± 11.3 to 9.3 ± 3.1, 12 months postoperatively, respectively. The total incidence of complications was 15.6%, including 2 cases of cage subsidence, 2 cases of ipsilateral hip flexor weakness, and 1 case of ipsilateral anterior thigh pain. All symptoms relieved or disappeared during follow-up. The fusion rate was 96.9%.

**Conclusions:**

Reoccurring discogenic LBP after discoblock should be considered as a suitable group for treatment by OLIF.

## Introduction

Intervertebral discogenic pain is the most common low back pain (LBP) and requires extensive medical attention. Discogenic LBP often persists and can severely affect the patient’s quality of life [1]. Degenerative discs are responsible for lumbar discogenic pain, which is defined as a disorder of the nucleus pulposus, rupturing of the annulus fibrosus, and injury of the cartilage endplate [2]. The treatment of patients with discogenic LBP continues to be a challenge. Medical treatment (nonsteroidal anti-inflammatory drugs [NSAIDs]), behavior management, psychotherapy, physical therapy, and rehabilitation are the primary treatment methods.

However, recrudescence is possible, and conservative treatment is sometimes ineffective in severe cases. Over the past few years, a variety of minimally invasive techniques have been developed to treat discogenic LBP [3–8], and discoblock is considered to be useful in relieving discogenic LBP [3]. However, this is ineffective in some patients, or pain recurs after discoblock. In these cases, open surgery is essential.

The pre-psoas approach, also known as oblique lumbar interbody fusion (OLIF), was introduced to gain access to the disc using an anterior approach between the aorta and psoas, instead of through the psoas itself, in order to avoid injury to the lumbar plexus. Coupled with direct visualization, where the intraabdominal structures are directly visualized during the approach, OLIF has shown several potential advantages, including avoidance of the lumbar plexus, and direct visualization of important structures such as sensory nerves, the ureter, great vessels, the lymphatics, and the sympathetic trunk [9, 10].

The OLIF method is considered a relatively safe approach. In recent years, good clinical results for OLIF have been reported, and OLIF techniques for discogenic LBP have also been reported [10–13]. However, no systematic study on this subject has been conducted yet and their reliability is somewhat controversial.

Therefore, the purpose of our study was to determine the efficacy of OLIF in the treatment of reoccurring discogenic LBP after discoblock.

## Materials and methods

### Inclusion criteria

Patients included had persistent LBP, which was suspected to be discogenic (such as high intensity zone (HIZ), Schmorl’s nodes, Modic changes Type I, etc.), and at least 3 months of conservative treatment (analgesia, bed rest, bracing, etc.) which had been unsuccessful. Besides, all patients underwent discography had LBP that was confirmed to be discogenic. The disc level was L2-5, which can undergo OLIF.

### Exclusion criteria

The exclusion criteria were as follows: leg pain; previous spinal surgery in the segment of interest; back pain due to other causes (such as recent vertebral body or endplate fracture, disc herniation, or spinal canal stenosis); spondylolisthesis; scoliosis; malignant tumors; severe kidney or liver disease; active infection; immunosuppression; allergy to local anesthetics, contrast media, or iodine; pregnancy; and chronic nicotine, alcohol, or drug abuse.

### Patients

**Fig. 1:**
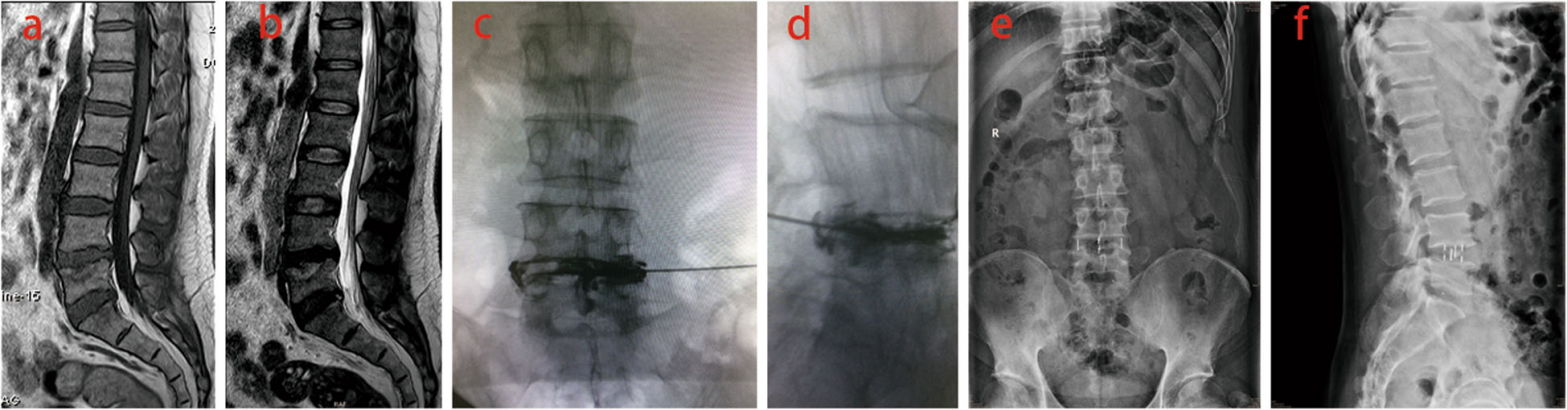
The vertebral body bone marrow surrounding painful Schmorl’s nodes was characterized by low T1 and high T2 signals in MRI (**a**, **b**). The patient experienced severe LBP and confirmed to be discogenic by discography (**c**, **d**). After the discoblock was administered, the patient complained LBP relieved. The patient had reoccurring pain and underwent OLIF after 8 months follow-up (**e**, **f**)

**Fig. 2:**
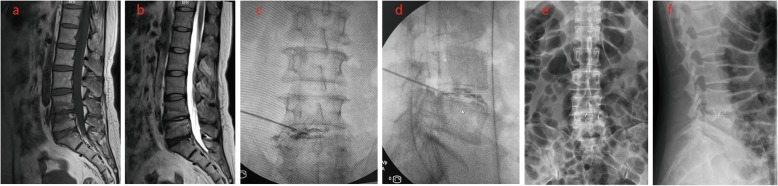
Modic changes type I was characterized by low T1 and high T2 signals in MRI on L4-5 endplate (**a**, **b**). The patient experienced severe LBP and confirmed to be discogenic by discography (**c**, **d**). After the discoblock was administered, the patient complained LBP relieved. The patient had reoccurring pain and underwent OLIF after 3 months follow-up (**e**, **f**)

**Fig. 3:**
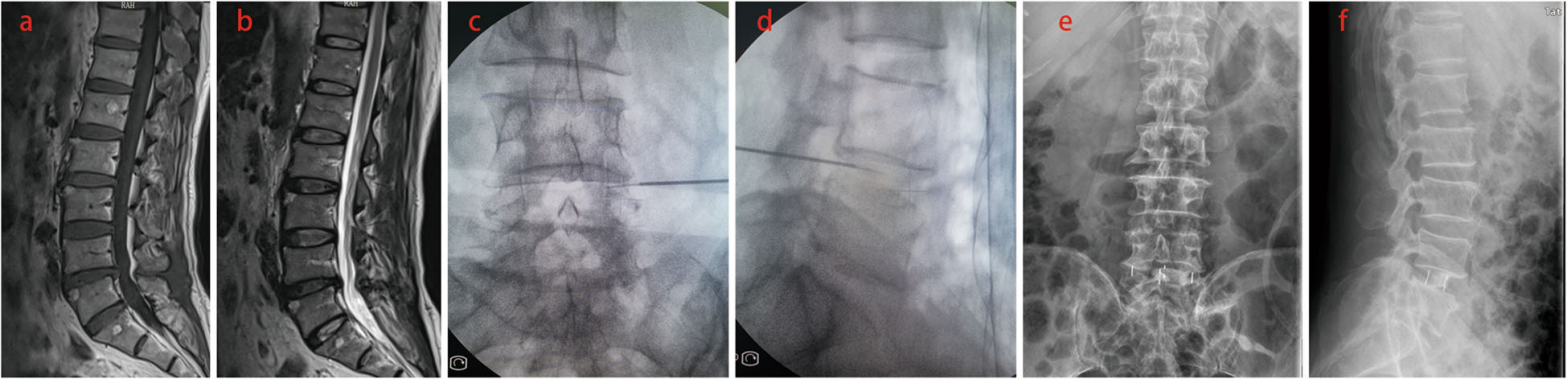
High intensity zone (HIZ) was characterized as a bright white signal on T2W images and low signal on T1 in the posterior annulus of the L4-5 disc (**a**, **b**). The patient experienced severe LBP and confirmed to be discogenic by discography (**c**, **d**). After the discoblock was administered, the patient complained LBP relieved. The patient had reoccurring pain and underwent OLIF after 2 months follow-up (**e**, **f**)

We included 108 patients (46 men and 62 women; mean age, 50.8 years; age range, 39–64 years) with LBP, which were suspected to be discogenic from August 2015 to August 2017. All patients underwent discography, and discoblock was given to positive patients whose LBP was confirmed to be discogenic. Discogenic LBP patients who had reoccurring pain after discoblock underwent OLIF (Figs. 1, 2, and 3).

Ethical approval was obtained from the Medical Ethics Committee of the hospital. Additionally, all patients gave written informed consent for their information to be stored in the hospital’s database and used for research.

### The criteria for stand-alone OLIF

No endplate damage during the operation, and postoperative flexion-extension radiograph appeared to be stable. Generally, we perform one-stage OLIF operation without pedicle screw-rod instrumentation if no endplate damage occurred during the operation. Besides, flexion-extension radiograph was evaluated postoperatively; if postoperative flexion-extension radiograph was shown to be unstable, a 2nd-stage pedicle screw fixation operation was performed.

### Imaging and clinical evaluation

Plain X-ray, MRI, and multi-slice computed tomography (CT) were performed to evaluate the main cause of LBP and to help to plan the treatment.

Perioperative parameters (operation time, blood loss, incision length, and length of hospital stay) and complications were recorded. Levels of serum creatine kinase were measured preoperation, and 1, 3, and 5 days after the operation. The visual analog scores (VAS) and Oswestry Disability Index (ODI) were assessed at preoperation, 1 week, and 1, 3, 6, and 12 months after surgery. The fusion rate was evaluated 12 months after the surgery.

### OLIF procedures

The patient was placed in a right-sided lateral decubitus position. Under C-arm control, anatomical surface of the disc in true lateral view was marked on the skin.

Standard preoperative preparation of the surgical field was done. For single-level fusion, a 2.5–4.5-cm skin incision was made centered in the projection of the target segment and parallel to external oblique muscle fibers.

The external oblique muscle, internal oblique muscle, and transverse abdominal muscle were dissected along the direction of their fibers with a blunt muscle-splitting technique. The retroperitoneal space was accessed by blunt dissection along the retroperitoneal fat tissue. The peritoneal sac was mobilized anteriorly. A periosteal detacher was used to gently push back the psoas muscle, and then a retractor was used to pull the psoas muscle to the dorsal side and pull the abdominal organs, together with the extraperitoneal fat, to the ventral side. Consequently, the intervertebral space of the lesion was exposed, and the tubular retractor system was docked. Special attention was given to the genitofemoral nerve, the sympathetic chain, and segmental blood vessels. C-arm was used to confirm the correct level. After discectomy, vertebral endplates were prepared and the subchondral bone was exposed. To achieve interbody fusion, the cage was packed with injectable graft (MIIG, Wright, UK). The absorbable suture was used to bind the cage to prevent the injectable graft from falling off. And the cage was inserted in a press fit fashion into the exposed disc spaces.

### Data analysis

Data were input into a Microsoft Excel spreadsheet (Microsoft, Redmond, Washington) and transferred to SPSS version 20.0 software (PASW, Statistics, IBM, USA). Quantitative results were expressed in terms of their mean and standard deviation. VAS and ODI at 1 week and at 1, 3, 6, and 12 months after the procedure were compared with one-way analysis of variance. The differences between different time points were compared with Fisher’s least significant difference test. A significance level of 0.05 was used, and *p* < 0.05 was considered significant, without multiple test adjustment.

## Results

### General characteristics

Of 108 patients, 89 were confirmed to have discogenic LBP, no spinal headache, discitis, intrathecal hemorrhage, arachnoiditis, etc. were found during the treatment of discography and discoblock, and 32/89 patients with reoccurring LBP pain after discoblock underwent OLIF. OLIF was performed 2 times at L2/3, 7 times at L3/4, and 23 times at L4/5. Twenty-eight patients were followed up for ≥ 1 year (mean, 16.3 months; range, 12–26 months). All 28 patients underwent stand-alone OLIF, and no patient underwent 2nd-stage pedicle screw fixation operation.

### Perioperative parameters

The OLIF operation lasted for 57–128 min, with a mean duration of 92 ± 34 mins. Blood loss during the operation was 20–70 ml, with a mean of 48 ± 15 ml. The mean incision length was 3.0 ± 0.6 cm, ranging from 2.4–4.1 cm. The average length of stay was 4.8 ± 1.9 days, ranging from 3 to 7 days. The serum level of creatinine kinase was 123.5 ± 48.1 IU/L before operation, 216.8 ± 82.1 IU/L 1 day after operation, 321.4 ± 37.5 IU/L 3 days after operation, and 161.4 ± 42.5 IU/L 5 days after operation. There was a higher level of serum creatine kinase at postoperative day one and day three (*P* < 0.05), but these differences did not persist at postoperative day 5 (*P* > 0.05).

### Clinical results

**Fig. 4:**
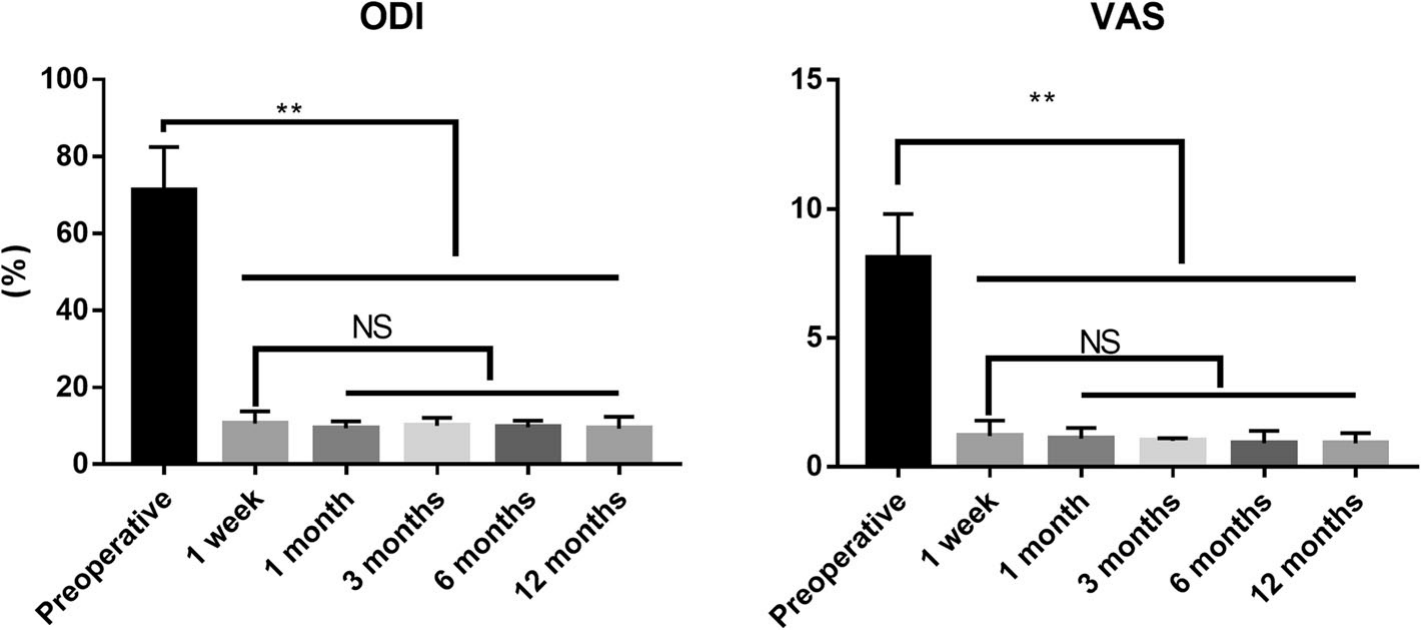
VAS and ODI scores decreased significantly after 1 week from the procedure (*p* < 0.05), but there were no significant differences between 1 week, and 1, 3, 6, and 12 months (*p* > 0.05) (**p < 0.01)

The VAS score decreased from 8.1 ± 1.7 (preoperatively) to 0.9 ± 0.4, and the ODI score decreased from 71.2 ± 11.3 (preoperatively) to 9.3 ± 3.1 12 months postoperatively. VAS and ODI scores decreased significantly after 1 week from the procedure (*p* < 0.05), but there were no significant differences between 1 week, and 1, 3, 6, and 12 months (*p* > 0.05) (Table 1 and Fig. 4).

**Table 1:** The preoperative and postoperative comparison of ODI and VAS for patients who underwent OLIF

	Preoperative	1 week	1 month	3 months	6 months	12 months	*F*	*P*
ODI (%)	71.2 ± 11.3	10.6 ± 3.2	9.4 ± 1.8	10.0 ± 2.1	9.6 ± 1.8	9.3 ± 3.1	2.41	*P* < 0.05
VAS	8.1 ± 1.7	1.2 ± 0.6	1.1 ± 0.4	1.0 ± 0.1	0.9 ± 0.5	0.9 ± 0.4	2.35	*P* < 0.05

### Intraoperative complications and fusion rate

The total incidence of complications was 15.6%, including 2 cases of cage subsidence, 2 cases of ipsilateral hip flexor weakness, and 1 case of ipsilateral anterior thigh pain. All symptoms were relieved or disappeared during follow-up. The fusion rate was 96.9%.

## Discussion

LBP is one of the most common public health concerns, which limits activity, causes significant disability, and creates a heavy social burden [14]. Internal disc disruption (IDD) characterized by degradation of the nucleus and disruption of the inner lamella of the annulus fibrosus is thought to be the major cause of chronic LBP [15–17]. The diagnosis of discogenic LBP due to IDD is difficult and controversial because of a lack of specific features. Recently, several studies reported that the presence of HIZ, Schmorl’s nodes, Modic changes type I in the affected disc or endplate on MRI scans contributed to the effective diagnosis of discogenic LBP [18–21]. Provocative discography, which aims to reproduce the patients’ symptoms by stimulating the suspicious disc but not the adjacent discs, is considered to be a main diagnostic test for discogenic LBP [17, 20, 21]. In our study, we used this technique for diagnosing discogenic LBP.

Even identified, discogenic LBP is difficult to treat. During the last few decades, minimally invasive techniques have been developed as an alternative to treat discogenic LBP. Discoblock is considered to be useful for relieving discogenic LBP. However, this is ineffective in some patients. For the patients who have reoccurring pain after discoblock, lumbar interbody fusion (LIF) is a surgical option that removes pain-generating compressive tissue, eliminates painful segmental motion, and restores sagittal balance [22].

There are several approaches through which LIF can be performed; traditional posterior lumbar interbody fusion can cause unavoidable damage to paraspinal back muscles, soft tissue, and the posterior bone structure of the lumbar spine. Moreover, it can expose neural elements in the spinal canal to iatrogenic injury. In the past few decades, many surgeons have explored various surgical approaches, focusing especially on minimally invasive techniques, to achieve lumbar fusion while avoiding complications caused by posterior lumbar interbody fusion.

Oblique lateral interbody fusion (OLIF) has emerged as a minimally invasive procedure for degenerative lumbar diseases intended to mitigate challenges experienced with trans-psoas approaches. The goal of the oblique trajectory is to access the interspace while avoiding disruption of the psoas and lumbosacral plexus.

In recent years, good clinical results of the OLIF have been reported, and OLIF techniques for discogenic LBP have also been reported [10–13]. However, no systematic study on this subject has been conducted yet. Therefore, in our study, we included 28 patients with reoccurring LBP pain after discoblock, who underwent OLIF. Our study assessed clinical results in terms of pain control and functional improvement. The results showed that patients with discogenic LBP refractory to conservative management and discoblock experienced a significant reduction in pain and functional improvement after OLIF. Showing satisfactory clinical outcomes in discogenic LBP patients, OLIF may be considered as an alternative surgical method to treat discogenic LBP. In the previous study [23], the radiographic and clinical outcomes of OLIF and TLIF for degenerative lumbar disease has been compared, which showed that the radiographic and functional outcomes and length of hospital stay were similar between the two groups, and the OLIF group showed advantages in operative blood loss and operative time.

Our study had several limitations. Firstly, the study was retrospective by design and had a small sample size. Secondly, the follow-up period was short, which may have limited the accuracy of the study. Thirdly, OLIF is not recommended for reoccurring discogenic LBP on L5/S1. In addition, these findings are based on a retrospective chart review, which lacked a comparison group. Further randomized studies with a longer follow-up period are needed to compare the OLIF approach with other approaches.

Nevertheless, this study suggests patients with reoccurring LBP pain after discoblock, who underwent OLIF with or without posterior pedicle screw-rod instrumentations, experienced a significant reduction in pain and functional improvement. The anterolateral minimally invasive oblique lumbar interbody fusion approach is a less-traumatic surgical technique for anterior lumbar fusion procedures. Discogenic LBP patients with reoccurring pain after discoblock should be considered as a suitable group for treatment by OLIF.

## Data Availability

The datasets used and analyzed during the current study are available from the corresponding author on reasonable request.

## Abbreviations

CT: Computed tomography
HIZ: High intensity zone
IDD: Internal disc disruption
LBP: Low back pain
LIF: Lumbar interbody fusion
NSAIDs: Nonsteroidal anti-inflammatory drugs
ODI: Oswestry Disability Index
OLIF: Oblique lumbar interbody fusion
VAS: Visual analog scores
